# Lanthipeptides from *Chitinophaga pinensis* re-potentiating β-lactams against metallo-β-lactamase-producing Enterobacterales *in vitro*

**DOI:** 10.1128/spectrum.00340-26

**Published:** 2026-07-24

**Authors:** Anastasia Lambropoulou, Jennifer Herrmann, Felix Deschner, Rolf Müller, Stathis D. Kotsakis

**Affiliations:** 1 Laboratory of Bacteriology, Hellenic Pasteur Institute, Athens, Greece; 2 Helmholtz Institute for Pharmaceutical Research Saarland, Helmholtz Center for Infection Research and Department of Pharmacy of Saarland University, Saarbrücken, Germany; JMI Laboratories, North Liberty, Iowa, USA

**Keywords:** metallo-β-lactamases, carbapenemases, lanthipeptides

## Abstract

During a screening of secondary metabolites produced by soil bacteria, we identified two lanthipeptides from *Chitinophaga pinensis*, pinensin A and pinensin B, that exhibit strong *in vitro* synergy with β-lactams against metallo-β-lactamase (MβL)-producing Enterobacterales. Both compounds restored meropenem sensitivity in 30 MβL-producing Enterobacterales to levels below the clinical breakpoint. Mechanistic analysis identified pinensins as high-affinity zinc binders, exhibiting nanomolar dissociation constants, explaining the synergistic effect. The properties of pinensins warrant their further evaluation as β-lactam potentiators and highlight specific microbial taxa as promising sources for metalloenzyme inhibitor discovery.

**IMPORTANCE:**

Resistance of clinical Enterobacterales to last resort drugs, such as carbapenems and newer cephalosporins, requires urgent measures. Production of metallo-β-lactamases is an important factor contributing to the problem, as there is no available therapeutic option against them. As with many of our currently utilized antibiotic classes, the environment has proven to be a valuable source of novel compounds, and naturally occurring inhibitors of metallo-β-lactamases may still await discovery. Herein, we show that such examples are two lanthipeptides produced by the soil bacterium *Chitinophaga pinensis,* which were able to counter the action of metallo-β-lactamases during the *in vitro* inhibition of clinical strains by β-lactams. The two compounds, previously identified as potent antifungal agents, exhibit strong binding and a degree of specificity for the enzymes’ cofactor (i.e., zinc), indicating that there is a potential for therapeutic applications.

## OBSERVATION

Healthcare-associated infections caused by multidrug-resistant Gram-negative bacteria are a major problem for contemporary medicine and have remained unsolved for nearly three decades. Production of metallo-β-lactamases (class B β-lactamases mainly of the B1 subclass; MβLs) by these strains is a significant factor contributing to this phenomenon, as these enzymes are able to hydrolyze the majority of β-lactams, which are the drugs of choice for such infections. Unlike their serine reactive counterparts—i.e., class A, C, and D β-lactamases—B1 subclass MβLs do not show preference for a specific β-lactam class and, with the exception of monobactams (e.g., aztreonam), can efficiently hydrolyze most of them (1). Moreover, due to their distinct reaction mechanism, they are not inactivated either by the suicide class A β-lactamase inhibitors (e.g., clavulanate, tazobactam, sulbactam) or the diazabicyclooctane class of broad-spectrum deactivators of serine reactive β-lactamases (e.g., avibactam and relebactam), which are currently in clinical use (2, 3). These characteristics accelerate the selection of MβL-producing strains due to their resistance to a great percentage of β-lactam-based chemotherapies administered in hospitals and the community (2). Indeed, the circulation of MβL-expressing mobile genetic structures in healthcare settings is steadily increasing, with the carrying strains causing worldwide epidemics (e.g., Enterobacterales producing NDM-type MβLs [2, 4]).

A non-toxic and efficient MβL inhibitor that could be used in combination with β-lactams would undoubtedly help alleviate the problem, at least temporarily. Two promising MβL inhibitors, xeruborbactam and taniborbactam, are currently under evaluation and will most likely enter clinical practice (5, 6), although enzyme variants exhibiting resistance to inhibition have already been described (7). Considering that the environmental reservoir remains largely unexplored for microbial metabolites active against MβLs, we initiated a screening program targeting molecules purified from soil bacteria. During our initial screening, we observed that two nearly identical compounds, pinensins A and B, belonging to class I lanthipeptides (8, 9) and isolated from the Gram-negative bacterium *Chitinophaga pinensis* (10), restored the sensitivity of NDM-1-producing *Escherichia coli* to β-lactams *in vitro*. Pinensins have not been reported to exhibit direct antibacterial activity but represent the first lanthipeptides shown to possess antifungal activity (10), although their exact mechanism of action remains unknown. Herein, we show that pinensins also exhibit specific *in vitro* synergy with β-lactams against MβL-producing Enterobacterales, restoring meropenem minimum inhibitory concentrations (MICs) below the clinical breakpoint through a zinc-binding mechanism.

## RESULTS AND DISCUSSION

### Specificity and potency of pinensins/β-lactams synergy against MβL-producing strains

Specificity of the observed synergy against MβL-producing strains was assessed by measuring β-lactam MICs against *E. coli* laboratory strains and *Klebsiella pneumoniae* clinical isolates expressing β-lactamases from the four molecular classes (A, C, D, and B1 class β-lactamases). Ampicillin, cefotaxime, and meropenem MICs were determined using the broth microdilution method in Mueller-Hinton medium in the absence and presence of either pinensin A or B at a constant concentration of 50 μM. To ensure that the potential effects were clearly observable, each β-lactamase class was assayed against antibiotics that are its preferred substrates. Bacteria producing serine-reactive β-lactamases (class A: CTX-M-15, KPC-2; class C: CMY-2; class D: OXA-656) retained their resistance levels against the tested β-lactams in the presence of pinensins, as the determined MICs were equal to or one dilution lower than those determined without the compounds (Table 1). In contrast, 50 μM of either pinensin increased the susceptibility of the MβL-producing strains (VIM-1, VIM-27, NDM-1, IMP-1) to the action of β-lactams, with MICs being reduced to ≤8 µg/mL (ampicillin), ≤0.5 µg/mL (cefotaxime), and ≤0.25 µg/mL (meropenem), respectively (Table 1). Hence, the synergistic effects of pinensins were specific to MβLs.

**TABLE 1:** Specificity of pinensins’ synergy with β-lactams against subclass B1 MβL-producing strains

Strain	β-lactamase	Enzyme class^*a*^	β-lactam	β-lactam MIC (µg/mL)		
				No compound	Pinensin A (50 µM)	Pinensin B (50 µM)
*E. coli* DH5α/pB-bla_VIM-27_	**VIM-27**	B1	Ampicillin	4,096	≤8	≤8
*E. coli* DH5α/pC9-bla_CTX-M-15_	**CTX-M-15**	A	Ampicillin	8,192	8,192	8,192
*E. coli* DH5α/pB-bla_CMY-2_	**CMY-2**	C	Ampicillin	1,024	1,024	2,048
*E. coli* TOP10/IncQ-pGU13	**OXA-656**	D	Ampicillin	2,048	2,048	2,048
*E. coli* DH5α/pB-bla_VIM-27_	**VIM-27**	B1	Cefotaxime	64	≤0.5	≤0.5
*E. coli* DH5α/pC9-bla_CTX-M-15_	**CTX-M-15**	A	Cefotaxime	256	256	128
*E. coli* DH5α/pB-bla_KPC-2_	**KPC-2**	A	Cefotaxime	4	4	2
*E. coli* DH5α/IncFII-p5171	**NDM-1**, OXA-1	B1	Cefotaxime	64	≤0.5	≤0.5
*K. pneumoniae* UoA17	**VIM-1**	B1	Meropenem	64	ND	≤0.25
*K. pneumoniae* LA28	**NDM-1**	B1	Meropenem	16	ND	≤0.25
*K. pneumoniae* 5742	**IMP-1**	B1	Meropenem	16	ND	≤0.25
*K. pneumoniae* E1253	**KPC-2**	A	Meropenem	64	ND	32

^*a*^ Enzyme class concerns the β-lactamase type (denoted with bold) that provided resistance to the assayed antibiotic.

**Fig 1:**
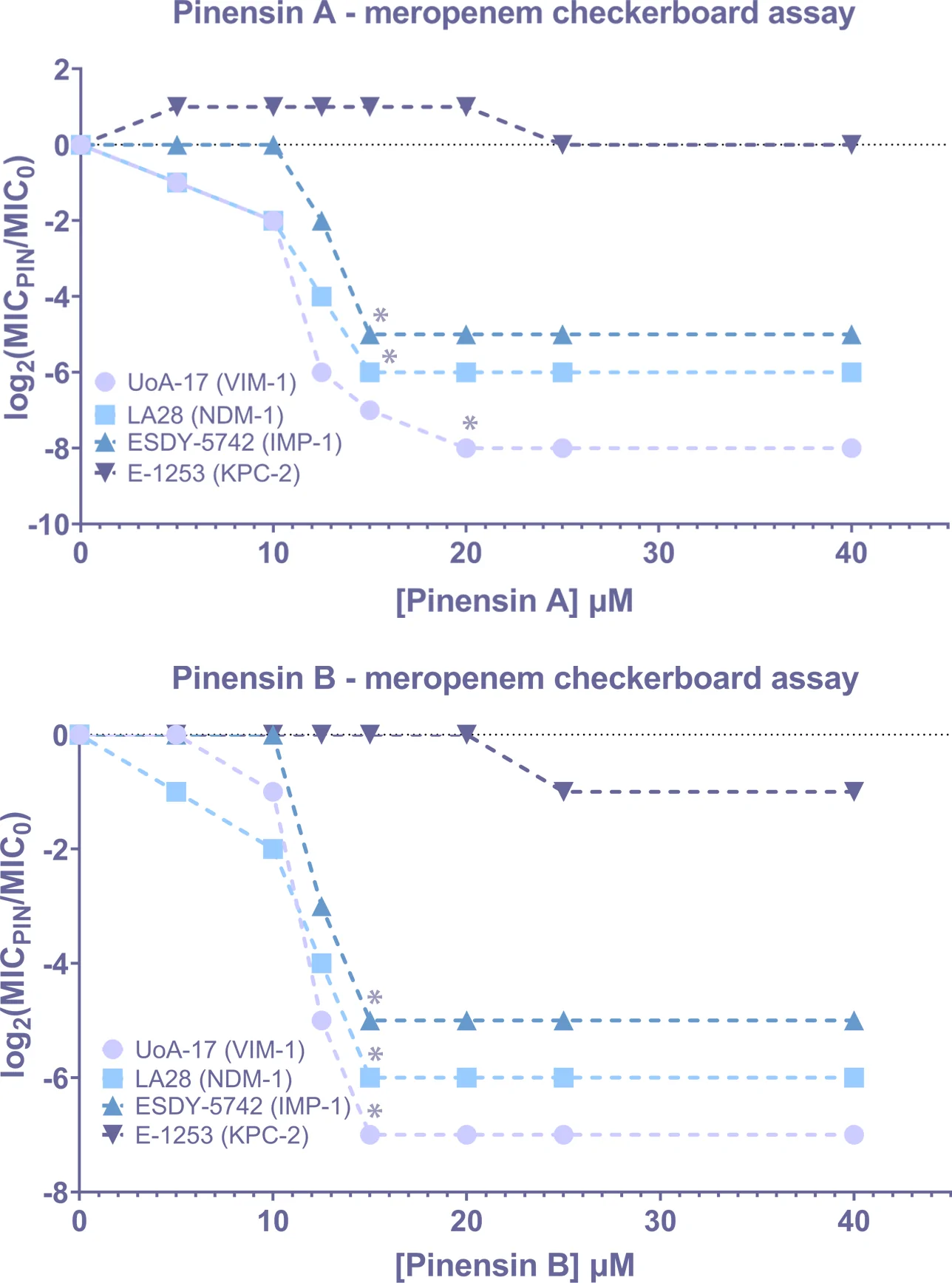
Checkerboard assays of synergistic effects between pinensins and meropenem against carbapenemase-producing *K. pneumoniae*. Doubling dilutions differences of meropenem MICs at each pinensin concentration (MIC_PIN_) compared to that determined in the absence of the compounds (MIC_0_) expressed as log_2_(MIC_PIN_/MIC_0_). The asterisks (*) in the graphs indicate that meropenem MIC at these concentrations was <0.25 µg/mL and hence there was a reduction that was higher than or equal to the denoted values.

The potency of the synergy was examined through checkerboard assays in which meropenem MICs against three clinical *K. pneumoniae* MβL producers were estimated in the presence of various pinensin concentrations (Fig. 1). A *K. pneumoniae* isolate producing the KPC-2 class A carbapenemase was also included as a control. The minimum pinensin concentration at which the effects on meropenem MICs against MβL producers became significant (>2 doubling dilutions) was 12.5 μM (Fig. 1). A concentration of 15 μM was sufficient to inhibit the growth of the MβL strains even at the lowest meropenem concentration tested (0.25 mg/L; MIC reduced by ≥5 doubling dilutions) for both pinensins (Fig. 1).

### *In vitro* efficacy of meropenem/pinensin combinations against MβL-producing Enterobacterales

The *in vitro* efficacy of the combinations against MβL-producing Enterobacterales was assessed by determining meropenem MICs against characterized strains producing VIM, NDM, and IMP enzymes, with the compounds maintained at a constant concentration of 15 μM. The collection comprised 30 isolates (VIM: *n* = 9, NDM: *n* = 10, IMP: *n* = 10, VIM/NDM: *n* = 1) belonging mainly to *Klebsiella pneumoniae,* as well as other Enterobacterales species (Table S1). All strains were isolated in clinical settings, except for the IMP producers, which were of environmental origin. In the majority of strains, pinensins reduced meropenem MICs to at or below 0.25 mg/L, even when baseline resistance was high (i.e., 16–128 mg/L; Table S1). Strains belonging to *Klebsiella aerogenes* and *Enterobacter cloacae* exhibited the lowest MIC reduction observed (2 doubling dilutions; Table S1), likely due to additional carbapenem resistance mechanisms (e.g., porin loss combined with overexpression of their chromosomal cephalosporinase). MIC percentiles (Table 2) suggested that pinensins at 15 μM restore the susceptibility of MβL-producing Enterobacterales to meropenem below the clinical breakpoint (sensitive ≤2 mg/L, resistant >8 mg/L; EUCAST Clinical Breakpoint Tables v. 15.0 [11]).

**TABLE 2:** *In vitro* efficacy of pinensins in restoring the susceptibility of 30 Enterobacterales strains producing VIM, NDM, or IMP type MβLs to meropenem expressed as MIC percentiles^*b*^

Isolates	No pinensin			15 μM Pinensin A			15 μM Pinensin B		
	MIC_50_	MIC_75_	MIC_90_	MIC_50_	MIC_75_	MIC_90_	MIC_50_	MIC_75_	MIC90
All (*n* = 30)	8	12	64	0.25	0.25	1	0.25	0.25	1
VIM (*n* = 10)^*a*^	4	64	128	0.25	0.25	0.5	0.25	0.25	0.5
NDM (*n* = 10)	8	16	128	0.25	0.5	8	0.25	1	8
IMP (*n* = 10)	4	8	8	0.25	0.5	2	0.25	0.5	2

^*a*^ One strain included in the VIM group co-produced the NDM-1 ΜβL.

^*b*^ Calculated in GraphPad’s Prism v8 as median values.

### Synergy was zinc-dependent

**Fig 2:**
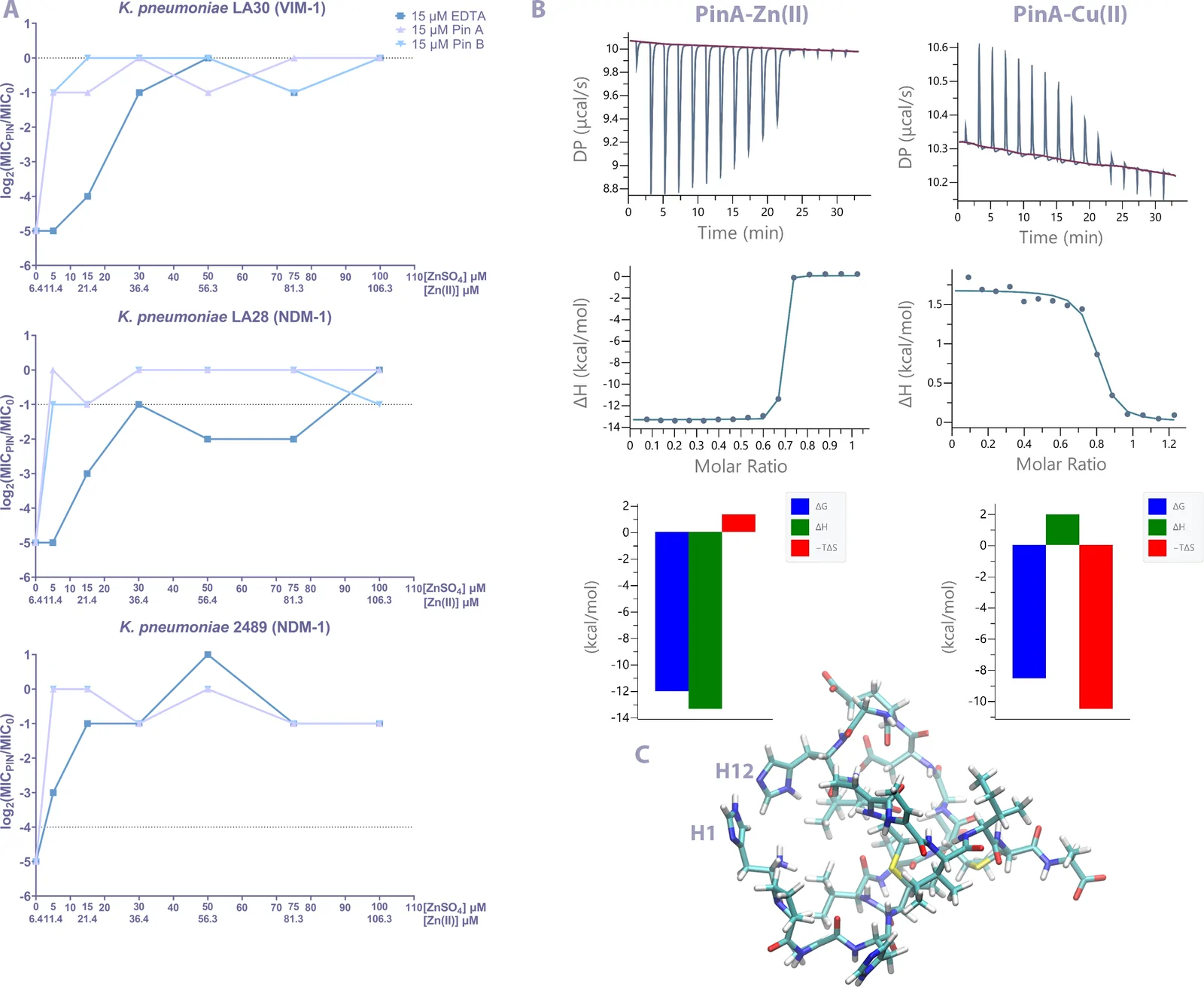
(**A**) Effect of Zn(II) on synergy between meropenem and pinensins or EDTA. Meropenem MICs in the presence of each inhibitory molecule were compared to those measured without a compound under various Zn(II) concentrations (secondary χ-axis). PinA, pinensin A; PinB, pinensin B. Dashed horizontal lines indicate the doubling dilution differences of the meropenem clinical breakpoint (8 μg/mL) compared to the MIC of the respective strains. (**B**) Isothermal titration calorimetry data obtained during interactions between pinensin A and Zn(II) (left panel) and pinensin A and Cu(II). Differential power (DP) curves and the calculated enthalpy (ΔΗ) showed that zinc binding was exothermic, while for copper it was endothermic. ΔG values indicated that for both metals, the binding was favorable, but zinc interacted with higher affinity, driven by enthalpy, indicating polar interactions. In contrast, during interactions with copper, the entropic term (−TΔS) was the factor contributing to the binding energy (lower graphs). Data were analyzed with the MicroCal PEAQ-ITC software. (**C**) A hypothetical structure of pinensin A obtained with geometry optimization using the PM3 method revealed that His1 and His12 may be in proximity in the folded molecule, forming a likely binding site for Zn(II).

Production of MβLs, and hence the resistance levels conferred by these enzymes, is highly affected by zinc availability, with low concentrations of the ion resulting in reduced MICs (12, 13). This is most likely due to incorrect folding and reduced stability of the enzymes in the periplasmic space under Zn(II) limiting conditions (14, 15). Therefore, we assessed whether the observed synergy between pinensins and β-lactams results from Zn(II) depletion rather than from direct inhibition of MβL function by pinensins in the periplasm. The role of Zn(II) concentration in synergy was evaluated through zinc sulfate (ZnSO_4_) supplementation of the medium (Mueller-Hinton broth containing 6.4 ± 0.2 µM zinc, as determined by flame atomic absorption spectroscopy) in the presence of 15 μM of each pinensin during meropenem MIC determinations against three MβL producers. An equal concentration of the chelating agent ethylenediaminetetraacetic acid (EDTA) was included for comparison (Fig. 2A). At ZnSO_4_ concentrations above 5 μM (i.e., [Zn(II)]_total_ = 11.4 μM), synergy was diminished with no reduction in meropenem MICs observed in the presence of 15 µM pinensin (Fig. 2A). In contrast, EDTA retained its potency at Zn(II) levels as high as 36.4 μM (Fig. 2A). These results pointed to a zinc-related mechanism of action for pinensins.

To assess whether pinensins can sequester zinc ions from the medium, we studied their direct interactions with Zn(II) and Cu(II) using isothermal titration calorimetry (ITC). The data indicated that both lanthipeptides bind Zn(II) with a low nanomolar dissociation constant (*K*_D_). In fact, pinensins exhibited a preference for this ion compared to Cu(II), as binding was 10-fold stronger and enthalpy rather than entropy driven (Fig. 2B; Table 3; Fig. S1). The latter finding pointed to polar interactions between lanthipeptides and Zn(II), most likely involving histidines at positions 1 and 12, as indicated by a hypothetical structure of pinensin A optimized through quantum mechanical calculations (Fig. 2C).

**TABLE 3:** Binding and thermodynamic constants characterizing the interaction of pinensins A and B with Zn(II) and Cu(II) measured through ITC

Complex	*N*	K_D_ (nM)	ΔH (kcal·mol^−1^)	ΔG (kcal·mol^−1^)	−TΔS (kcal·mol^−1^)
PinA+Zn(II)	0.675 ± 0.026	3.53 ± 2.84	−13.45 ± 0.07	−11.65 ± 0.49	1.81 ± 0.66
PinA+Cu(II)	0.747 ± 0.027	420 ± 175	1.82 ± 0.21	−8.73 ± 0.25	−10.55 ± 0.07
PinB+Zn(II)	0.738 ± 0.031	6.34 ± 4.19	−13.45 ± 0.21	−11.3 ± 0.42	2.18 ± 0.25
PinB+Cu(II)	0.646 ± 0.080	129 ± 21.9	1.51 ± 0.11	−9.42 ± 0.11	−10.9 ± 0.0

### Conclusions

Based on our observations, pinensins are an example of naturally occurring compounds capable of restoring the activity of β-lactams against MβL-producing Enterobacterales. Although only a handful of such molecules have been described (16, 17), their occurrence in soil microbial communities may not be rare and could also be linked to other biological effects (e.g., growth inhibition of fungi in the case of pinensins [10]). Among these compounds, the fungal secondary metabolite aspergillomarasmine A (AMA) has been extensively studied (16, 18, 19). The molecule exerts its action through metal chelation and exhibits *in vitro* synergy with β-lactams against MβL-producing Gram-negative bacteria. The *in vivo* efficacy of AMA in combination with meropenem has also been established in a murine model of *K. pneumoniae* infection (16). Moreover, it has been suggested that the molecule accumulates in *E. coli* cells, indicating efficient uptake and direct enzyme inhibition (18).

In contrast to small chelating agents, the molecular mass of pinensins (~2 KDa) likely precludes passive crossing of the outer membrane through porins. Therefore, since it is currently unknown whether pinensin-specific outer membrane transport mechanisms are present in Enterobacterales, the observed phenotype may result from extracellular zinc depletion rather than direct competition for the enzyme cofactor in the periplasm. Further mechanistic studies are therefore needed.

Furthermore, as previous studies have shown moderate cytotoxic effects *in vitro* against various human cell lines (10), *in vivo* studies of pinensins are warranted to assess their safety before evaluating their efficacy in infection models. Although the biological role of pinensins may involve transition metal scavenging, our preliminary data indicated a preference for zinc, at least compared to copper. The above suggests that their potent synergistic effects with meropenem against ΜβL-producing Enterobacterales strains may be relatively specific and that the molecules may not interfere with the homeostasis of other metals *in vivo*.

Nonetheless, the fact that the producing organism belongs to *Chitinophagia*—a group cohabiting the same ecological niches and closely associated with bacteria implicated in the evolution of MβLs, namely *Cytophagia* and *Myxococcia* (20)—highlights the potential for identifying additional inhibitors through targeted screening of related strains.

## MATERIALS AND METHODS

### Bacterial strains and plasmids

Laboratory strains utilized as hosts for the recombinant expression of β-lactamases were *E. coli* DH5α [fhuA2 lac(del)U169 phoA glnV44 Φ80′ lacZ(del)M15 gyrA96 recA1 relA1 endA1 thi-1 hsdR17] and *E. coli* TOP10 [F-mcrA (mrr-hsdRMS-mcrBC) 80lacZ M15 lacX74 recA1 ara 139 (ara-leu)7697 galU galK rpsL (StrR) endA1 nupG]. CMY-2, VIM-27, and KPC-2 β-lactamases were expressed from previously described constructs of the pBC-SK(±) vector (21–23), while CTX-M-15 was expressed from a pC9 derivative (24). The wild-type plasmids InQ-pGU13 and IncFII-p5171 were utilized as sources of the OXA-656 and NDM-1 enzymes, respectively (25, 26).

Wild-type isolates used in the study were isolated either from clinical settings in Greece or from environmental sources (IMP producers), and their β-lactamase content had been fully characterized (Table Table S1 [27–29]). Thirty MβL-producing isolates were utilized, belonging to *Klebsiella pneumoniae* (*n* = 16), *Enterobacter* cloacae (*n* = 2), *Klebsiella aerogenes* (*n* = 4), *Proteus mirabilis* (*n* = 2), *Escherichia coli* (*n* = 2), *Serratia liquefaciens* (*n* = 1), *Providencia stuartii* (*n* = 1), *Kluyvera georgiana* (*n* = 1), and *Citrobacter freundii* (*n* = 1). Furthermore, a KPC-2-producing *K. pneumoniae* isolate was included for comparison. *K. pneumoniae* strains harboring identical β-lactamase content were confirmed to be unique through multilocus sequence typing and restriction fragment length polymorphism analysis of genomic DNA using pulsed-field gel electrophoresis.

### β-lactam susceptibility and synergy assays

MICs of β-lactams were determined by microdilution in Mueller-Hinton (MH) broth (Sharlau, Spain) according to EUCAST guidelines (11). The β-lactams used were ampicillin (ampicillin sodium; Sigma-Aldrich, USA), cefotaxime (cefotaxime sodium; Alpha Aesar, USA), and meropenem (meropenem trihydrate, Sigma-Aldrich, USA). Pinensins A and B were previously isolated and purified from the fermentation broth of a *Chitinophaga pinensis* strain (10).

The effects of pinensins on β-lactam resistance were assessed by measuring β-lactam MICs in the absence and presence of the compounds, which were tested either at a constant concentration of 50 μM (initial experiments) or 15 μM (*in vitro* efficacy assays against ΜβL-producing wild-type strains), as well as at varying concentrations of 40, 25, 20, 15, 12.5, 10, and 5 μM during checkerboard assays. In the latter assays, the results were expressed as doubling dilution differences between the MICs measured at each pinensin concentration and those measured in the absence of the compounds.

The effects of zinc on the synergistic activity of pinensins and β-lactams against MβL-producing Enterobacterales were assessed by measuring meropenem MICs in the presence of 15 μM of each compound and varying concentrations of zinc sulfate (Serva, Germany), with results again expressed as doubling dilution differences relative to control assays lacking pinensins. Parallel assays in which pinensins were replaced by EDTA were also performed.

As working solutions of pinensins were prepared in dimethyl sulfoxide, all MIC determinations were performed in the presence of 2% vol/vol organic solvent.

### Zinc content determinations

The zinc content of the MH broth used in MIC measurements was determined by flame atomic absorption spectrometry (FAAS). Approximately 1.0 mL of each sample, accurately weighed, was digested with 6 mL of nitric acid (65% Suprapur; Merck, Germany) in a microwave digestion oven (Anton Paar Multiwave Go, Austria). Determinations were carried out using a Varian SpectrAA 200 instrument (Mulgrave, Australia).

### Isothermal titration calorimetry

The interactions of pinensins A and B with Zn(II) and Cu(II) were studied using ITC. Experiments were performed using a MicroCal PEAQ-ITC instrument (Malvern Panalytical, UK) equipped with a 200 µL cell and a 40 µL syringe. Samples were prepared in fresh 100 mM Tris/HCl, pH 7.5. In the standard setup, 100 µM compound was loaded into the cell (ligand), while 500 µM ZnSO_4_ (or 600 µM CuSO_4_) was filled in the syringe (analyte). Salt-buffer titrations were performed to correct for dilution heat. The stirring speed was set to 750 rpm, and the analyte was injected through one initial injection of 0.4 µL, followed by 15 injections of 2.5 µL with 120 s spacing to allow baseline separation. All experiments were performed in duplicate at 25°C. The cell and stirrer were thoroughly cleaned using methanol, 10% Contrad 70, and Milli-Q water between runs using the automated cleaning module. Data analysis was performed using the MicroCal PEAQ-ITC software.

### Pinensin A modeling

A three-dimensional model of pinensin A was built using the GaussView software, and its geometry was optimized in the gas phase using the semi-empirical Parametric Method 3 (PM3) implemented in Gaussian 09, with connectivity being explicitly specified. The system had an overall charge of −4 and a singlet spin state. Optimizations were performed using the default convergence criteria of Gaussian. The optimized model, representing a local minimum on the molecule’s potential energy landscape, was visualized using VMD v1.9.3 software (30).

## Data Availability

All data generated during this study are included in this paper and its Supplemental material.

## SUPPLEMENTAL MATERIAL

Supplemental material spectrum.00340-26-s0001.docx Table S1 and Fig. S1.

ASM does not own the copyrights to Supplemental Material that may be linked to, or accessed through, an article. The authors have granted ASM a non-exclusive, world-wide license to publish the Supplemental Material files. Please contact the corresponding author directly for reuse.
