# Supplementary material for: Lanthipeptides from Chitinophaga pinensis re-potentiating β-lactams against metallo-β-lactamase-producing Enterobacterales in vitro
Source: Microbiol Spectr. 2026 Jul 24;14(9):e00340-26. doi: 10.1128/spectrum.00340-26 (PMC13532221; doi:10.1128/spectrum.00340-26)
Supplement: Supplementary file 1 [file spectrum.00340-26-s0001.docx]

**Supplemental Table 1:** Synergy of pinensins A and B with meropenem against a collection of MβL-producing Enterobacterales.

**Strain	β-lactamases	Meropenem MIC (μg/ml)**

**-------------------------------------------------------------------------**

**No compound	Pinensin A (15 μΜ)	Pinensin B (15 μM)**

**VIM type ΜβL producers**

***K. pneumoniae* UOA17	VIM-1**	64	0.5	≤0.25

***K. pneumoniae* E2681	VIM-27**	64	≤0.25	≤0.25

***K. pneumoniae* LA30	VIM-1**	8	≤0.25	≤0.25

***K. pneumoniae* TZ52/531	VIM-1**	4	≤0.25	≤0.25

***K. pneumoniae* IK14	VIM-1/**CMY-2 type	0.5	≤0.25	≤0.25

***K. pneumoniae* LAR26/I	VIM-1/NDM-1**	4	≤0.25	≤0.25

***Ent. cloacae* SEC-5	VIM-1/**SHV-5	1	≤0.25	≤0.25

***P. mirabilis* EUG91	VIM-1/**VEB-1	≤0.25	≤0.25	≤0.25

***P. stuartii* EUG323	VIM-1**	4	≤0.25	≤0.25

***S. liquefaciens* E815	VIM-1**	128	≤0.25	≤0.25

**NDM type ΜβL producers**

***K. pneumoniae* LA26	NDM-1/**CTX-M-15	16	≤0.25	1

***K. pneumoniae* LA27	NDM-1/**CTX-M-15	8	0.5	1

***K. pneumoniae* LA28	NDM-1/**CTX-M-15	16	≤0.25	≤0.25

***K. pneumoniae* LA31	NDM-1/**CTX-M-15	16	≤0.25	1

***K. pneumoniae* LA33	NDM-1/**CTX-M-15	4	≤0.25	≤0.25

***K. pneumoniae* 2489	NDM-1**	128	8	8

***K. pneumoniae* ALX71	NDM-1/**CTX-M-15	8	≤0.25	≤0.25

***K. pneumoniae* ALX73	NDM-1/**CTX-M-15	8	≤0.25	≤0.25

***K. aerogenes* ESDY-7448	NDM-1**	8	2	2

***K. aerogenes* ESDY-7362	NDM-1**	8	≤0.25	≤0.25

**IMP type MβL producers**

***K. pneumoniae* ESDY-5742	IMP-1**	8	≤0.25	≤0.25

***K. pneumoniae*** **1733m2	IMP-4**	8	≤0.25	≤0.25

***E. coli* 1543m1	IMP-4**	4	≤0.25	≤0.25

***E. coli* 1586m1	IMP-38**	1	≤0.25	≤0.25

***Ent. cloacae* 1537M	IMP-4**	8	2	2

***K. aerogenes* 1548M	IMP-4**	8	1	1

***K. aerogenes* 1659M2	IMP-4**	2	≤0.25	≤0.25

***Klu. georgiana* 1545M	IMP-4**	1	≤0.25	≤0.25

***C. freundii* 1643m2	IMP-38**	4	≤0.25	≤0.25

***P. mirabilis* 1539P	IMP-4**	8	≤0.25	≤0.25

**Supplemental Figure 1:** Isothermal titration calorimetry data of the interactions of pinensin A and pinensin B with ZnSO_4_ and CusO_4_.
